# BINGO: a blind unmixing algorithm for ultra-multiplexing fluorescence images

**DOI:** 10.1093/bioinformatics/btae052

**Published:** 2024-01-30

**Authors:** Xinyuan Huang, Xiujuan Gao, Ling Fu

**Affiliations:** Britton Chance Center for Biomedical Photonics, Wuhan National Laboratory for Optoelectronics, Huazhong University of Science and Technology, Wuhan 430074, China; MoE Key Laboratory for Biomedical Photonics, Huazhong University of Science and Technology, Wuhan 430074, China; Advanced Biomedical Imaging Facility, Huazhong University of Science and Technology, Wuhan 430074, China; Britton Chance Center for Biomedical Photonics, Wuhan National Laboratory for Optoelectronics, Huazhong University of Science and Technology, Wuhan 430074, China; MoE Key Laboratory for Biomedical Photonics, Huazhong University of Science and Technology, Wuhan 430074, China; Advanced Biomedical Imaging Facility, Huazhong University of Science and Technology, Wuhan 430074, China; Britton Chance Center for Biomedical Photonics, Wuhan National Laboratory for Optoelectronics, Huazhong University of Science and Technology, Wuhan 430074, China; MoE Key Laboratory for Biomedical Photonics, Huazhong University of Science and Technology, Wuhan 430074, China; Advanced Biomedical Imaging Facility, Huazhong University of Science and Technology, Wuhan 430074, China; School of Biomedical Engineering, Hainan University, Haikou 570228, China; School of Physics and Optoelectronics Engineering, Hainan University, Haikou 570228, China; Optics Valley Laboratory, Wuhan 430074, China

## Abstract

**Motivation:**

Spectral imaging is often used to observe different objects with multiple fluorescent labels to reveal the development of the biological event. As the number of observed objects increases, the spectral overlap between fluorophores becomes more serious, and obtaining a “pure” picture of each fluorophore becomes a major challenge. Here, we propose a blind spectral unmixing algorithm called BINGO (Blind unmixing via SVD-based Initialization Nmf with project Gradient descent and spare cOnstrain), which can extract all kinds of fluorophores more accurately from highly overlapping multichannel data, even if the spectra of the fluorophores are extremely similar or their fluorescence intensity varies greatly.

**Results:**

BINGO can isolate up to 10 fluorophores from spectral imaging data for a single excitation. nine-color living HeLa cells were visualized distinctly with BINGO. It provides an important algorithmic tool for multiplex imaging studies, especially in intravital imaging. BINGO shows great potential in multicolor imaging for biomedical sciences.

**Availability and implementation:**

The source code used for this paper is available with the test data at https://github.com/Xinyuan555/BINGO_unmixing

## 1 Introduction

In order to systematically understand the structure and function of organisms in different scenarios, multicolor imaging is usually used to observe the quantity, distribution, behavior, and interaction of multiple objects in organisms at the same time (Binnewies *et al.* 2018, Anderson and Simon 2020, Zhou *et al.* 2022, Yoon *et al.* 2023). Spectral imaging is currently the most commonly used method for multicolor imaging, through dispersion devices (such as prisms or gratings) and array detectors can collect fluorescence signals in the visible spectrum (Zimmermann *et al.* 2014, Sanderson 2019, Rehman and Qureshi 2021, Wang *et al.* 2023). With the development of devices and systems, the unit bandwidth of each channel of spectral imaging can now be set as low as about 9 nm, and the spectral resolution can reach 20 nm (Gao and Smith 2015, Bares *et al.* 2020). However, with the increase in the number of observation objects, the number of fluorophores that need to be used as detection labels also increases, and the spectral overlap between fluorophores will become more and more serious, and it is impossible to extract every observation object solely by the hardware. Spectral unmixing is a data processing process specifically dealing with such problems. In order to cope with the increasingly complex multitarget simultaneous observation requirements, more and more spectral unmixing algorithms have been developed (Maric *et al.* 2021). The existing algorithms can be divided into two categories: reference spectrum-based unmixing (Zimmermann *et al.* 2014, Rakhymzhan *et al.* 2017) and blind unmixing (Seo *et al.* 2022). Linear unmixing or supervised classification based on reference spectra has been the most commonly used method for spectral resolution for many years. The limitation of its application is that it needs to obtain reliable reference spectra, which greatly increases the complexity of the experiment, and complex physiological or pathological environment further increases the difficulty of obtaining reference spectra. Therefore, spectral resolution methods based on blind sources have gradually developed in recent years.

When we want to make a breakthrough in the number of multicolor imaging, we face the problems of high crosstalk between spectra, large intensity differences between fluorophores caused by the fluorophore's own properties, and difficulty in obtaining reference spectra. Researchers prefer to compress the bandwidth to acquire purer images directly in practical spectral imaging. In this case, the number of channels increases, but the bandwidth is too narrow, and the collected fluorescence signal is weak, leading to low signal-to-noise ratio (SNR), low contrast, and low-resolution images. Although an algorithm called HyU proposed a fast and effective noise reduction method for the pre-processing of spectral resolution, which is of great significance for image unmixing with low SNR, but in vivo imaging only stops at low scattering media such as zebrafish (Cutrale *et al.* 2017, Chiang *et al.* 2023). Therefore, in order to obtain higher-quality multicolor images, it is necessary to limit the number of channels to ensure sufficient bandwidth. Mathematically, when the number of channels equals the number of fluorophores, the un-mixing problem belongs to the determined system (the number of equations is equal to the number of variables), and a unique and correct solution can be obtained in principle (Zimmermann 2005). However, few unmixing algorithms have extracted accurate results under this condition (Supplementary Fig. S1). On the other hand, the emission spectra and fluorescence intensity of fluorophores are not only dependent on the structure of the fluorescence molecules but are also sensitive to the environment in which they are embedded (Ueno and Nagano 2011, Seo *et al.* 2022), and spectral shift in highly heterogeneous specimens is common. These reference spectra-based algorithms cannot deal with situations like that. A SIMI algorithm based on supervised classification was proposed, which can be applied even to the spectral unmixing of the overdetermined system, but it has failed to achieve a blind solution so far (Rakhymzhan *et al.* 2017, Rakhymzhan *et al.* 2021). PICASSO (Seo *et al.* 2022) is the only algorithm that can achieve blind unmixing with an equal number of fluorophores and channels, but the results are not satisfactory when the fluorescence intensity of fluorophores in the sample is very different. NMF algorithm is a common method in blind source spectral unmixing (Garbacik *et al.* 2018, Jiménez-Sánchez *et al.* 2019, Rossetti *et al.* 2019), which can reconstruct the original image matrix into a series of nonnegative base images with pure additive mixing, and has strong explanatory properties. However, the current NMF-RI (Jiménez-Sánchez *et al.* 2019), SSASU (Rossetti *et al.* 2019), and other algorithms still need the reference spectrum as a weak label, and the accuracy and robustness of the blind algorithm are still limited. In the case of an absolutely blind solution, existing unmixing algorithms have not been able to achieve separation of fluorophores greater than five colors in a single excitation round, which is currently a challenge.

In this article, we proposed a blind unmixing algorithm, which we named BINGO (Blind unmixing via SVD-based Initialization Nmf with project Gradient descent and spare cOnstrain). BINGO uses nonnegative matrix factorization (NMF) (Lee and Seung 1999) as a framework to extract endmembers from highly crosstalked multichannel images while estimating fluorophores' spectra. At the beginning of the factorization, BINGO uses a singular value decomposition (SVD)-based initialization (Boutsidis and Gallopoulos 2008) to predict the spectral shape of the fluorophores contained in the sample, eliminating the randomness of the decomposition results and achieving a successful blind solution. The sparsity constraint (Hoyer 2003) in the objective function and the projected gradient descent (Lin 2007) in BINGO help NMF to maintain a specific sparsity of feature matrix during the iteration, guiding the iteration toward reducing image crosstalk. By improving the multiple implementation process, BINGO is optimized for robustness, speed, and especially the completeness in absolutely blind unmixing so that it can be achieved in multicolor imaging simply and effectively. With the help of BINGO, pure and high-quality images of each target can be obtained without reference spectra from images with high crosstalk even that 10 times intensity difference between fluorophores. BINGO currently performs fluorophore extraction of up to eleven fluorophores with single excitation. Successfully applied to 10-color standard fluorescent beads and nine-color live HeLa cells. BINGO can obtain accurate targets no matter when the number of fluorophores increases or when the difference in fluorophore intensity increases, which is superior to other existing algorithms in ultra-multiplexed fluorescence imaging, providing a quantitative multidimensional analysis tool for biomedical imaging research.

## 2 Materials and methods

### 2.1 NMF unmixing

**Figure 1.**
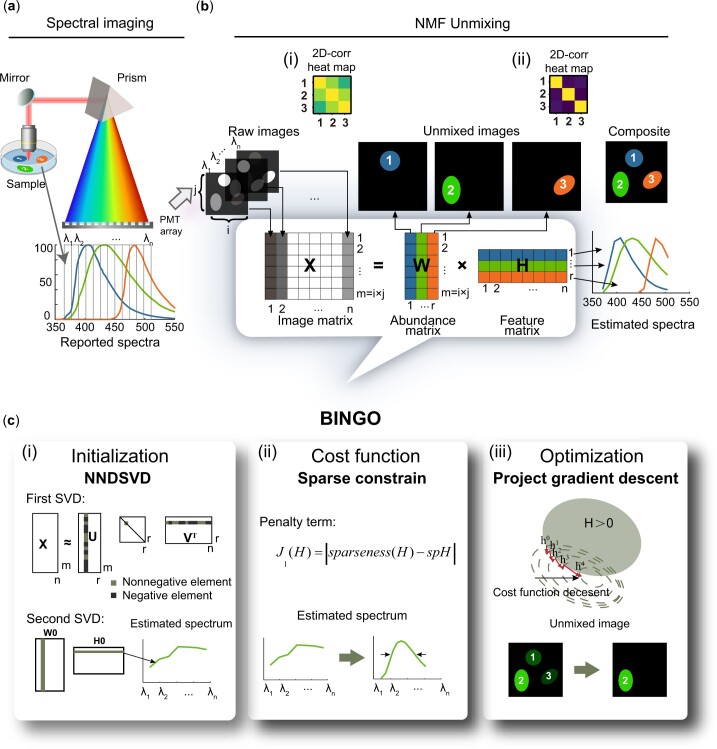
Graphical representation of the BINGO. (a) Spectral imaging of multiple fluorophores. Fluorescence signal from three fluorophores was dispersed by a prism and then collected by a PMT array. The reported emission spectra of three fluorophores and the channel setting are at the bottom. (b) Schematic principle of NMF unmixing. Raw images of n channels were flattened to a two-dimensional image matrix and then the image matrix was factorized into the abundance matrix W and feature matrix H respectively. Unmixed images can be obtained after rearranging the abundance matrix and the feature matrix represents the estimated spectra of all these fluorophores in the sample. The 2D-corr heat map in (bi) can show the crosstalk between different channels. (bii) After unmixing, crosstalk should be removed and the maximum value only appears on the diagonal of the heat map, which means that the image obtains a larger value only when the 2D-correlation coefficient is calculated with itself. (c) Essential parts of BINGO. (ci) BINGO uses NNDSVD initialization to estimate the spectra rapidly. (cii) Sparse constrain in the objective function limit the width of estimated spectra. (ciii) Project gradient descent of feature matrix H leads the iterations toward reducing image crosstalk.

BINGO is an image processing method applied to multichannel images to extract pure endmember from highly crosstalk images. Taking spectral imaging as an example (Fig. 1a), a sample labeled with multiple fluorophores (typ. N_fluo_ = r) is excited by a suitable light source. The fluorescence is collected by an objective, dispersed by optics such as a prism or a grating, and finally collected by a detector array (typ. PMT array) with n channels, and then the raw image of n channels is obtained. BINGO uses NMF as a framework, as shown in Fig. 1b and Supplementary Note 1. The first step of NMF unmixing is to flatten the image stack: the grayscale values corresponding to each pixel in an image of size i×j are arranged into column vectors in a consistent order, and the n-channel image stack is then downscaled into a two-dimensional image matrix X with a scale of m×n, m=i×j. The number of fluorophores in the sample is r, r≤min⁡(m, n), which satisfies the condition of the determined system to obtain the only correct solution. NMF is to find two nonnegative real matrices$\;W\in R^{m\times r}\;a\mathrm{nd}\;H\in R^{r\times n}$, thus describing the image matrix X using W and H,

(1) X≈WH

The form is consistent with the linear mixing model of the fluorescence signal, and both the low-rank approximation and the nonnegativity of NMF are well suited for spectral unmixing. The factorization matrices W and H are called the abundance and feature matrices, respectively. The abundance matrix W, as the name suggests, represents the spatial concentration distribution of each fluorophore, and the corresponding image of each fluorophore is obtained after rearranging the matrix to an image; the feature matrix H reflects the intensity distribution of each fluorophore in each channel, corresponding to its estimated spectrum.

BINGO uses an SVD-based initialization called nonnegative double singular decomposition (NNDSVD) to predict the spectra of the fluorophores contained in the sample at the beginning of the factorization, which eliminates the randomness of the decomposition results and thus enables highly robust blind solutions. The sparsity constraint in the objective function and the optimization strategy with projected gradient descent help the factorization to guide the iterations in the direction of reducing image crosstalk. In this paper, to test the feasibility of BINGO and to evaluate the performance of the algorithm, we design a series of experiments to compare BINGO with linear unmixing (LU) (Zimmermann 2005), similarity comparison (SIMI) (Rakhymzhan *et al.* 2017), semi-blind algorithm NMF-RI (Jiménez-Sánchez *et al.* 2019), and blind solution algorithm: NMF with random initialization (nNMF) (Berry *et al.* 2007) and PICASSO (Seo *et al.* 2022).

### 2.2 NNDSVD initialization

As described in Fig. 1ci, NNDSVD initialization was used to estimate the spectra rapidly. NMF based on SVD initialization can reduce the error quickly with faster convergence during iterative optimization and can help converge to the global optimal solution (Lee and Seung 1999, Berry *et al.* 2007, Esposito 2021). In addition, as a basic process in linear algebra, SVD does not contain random processes, so it contributes to robust of BINGO. The SVD initialization provides the basis for the orthogonality of the two matrices. When the SVD of the image matrix is performed, the following is obtained.

(2) $$X=U\Sigma V^{T}$$

(3) $$\begin{array}{c} X={\;\sigma }_{1}u_{1}{v_{1}}^{T}+{\;\sigma }_{2}u_{2}{v_{2}}^{T}+\cdots +{\;\sigma }_{r}u_{r}{v_{r}}^{T}\\ \Sigma =\left[\begin{array}{ccc} {\;\sigma }_{1} & 0 & 0\\ 0 & \ddots & 0\\ 0 & 0 & {\;\sigma }_{r} \end{array}\right],\;{\;\sigma }_{1}\geq {\;\sigma }_{2}\geq \cdots \geq {\;\sigma }_{r} \end{array}$$

The core idea of SVD is to recover the original matrix with as few singular values as possible with orthogonal bases. As described in Eq. 2, SVD decomposes the image matrix into three matrices, where the left singular matrix and the right singular matrix are unitary matrix that are mutually orthogonal, Σ is the diagonal matrix, and the singular values of the image matrix are on the diagonal. The biggest problem faced when applying SVD to NMF initialization is that the resulting left singular matrix U and right singular matrix V are not always nonnegative, and it is necessary to correct all elements of the matrix to nonnegative values using appropriate methods while maintaining the orthogonality of W and H as much as possible. The NNDSVD allows to be easily combined with existing NMF algorithms by correcting nonnegative elements while maximizing data integrity and orthogonality between two matrices through two low-rank approximations (Fig. 1c). Algorithm 2 (Supplementary Note 2) shows the steps of NNDSVD Initialization. The initialization values W0 and H0 are output by entering the image matrix X and the number of fluorophores to be solved.

### 2.3 Sparse constraint in objective function

Considering that the half height full width of the fluorophore spectrum is certain and each fluorescence signal is mainly concentrated in some individual channels, we implement a sparse constraint for H

(4) $$f\left(W,\;H\right)={\left\|X-\mathrm{WH}\right\|}_{F}^{2}+\alpha J_{1}\left(H\right),$$

(5) $$\mathrm{sparseness}\left(H\right)=\;\frac{\sqrt{n}-L_{1}/L_{2}}{\sqrt{n}-1},$$

(6) $$L_{1}=\;\sum_{i=1}^{n}H_{i},L_{2}=\;\sqrt{\sum_{i=1}^{n}{H_{i}}^{2}},$$

where F is the L2 norm, α is the weighting factor, which is an empirical value, J_1_(H) is the sparsity penalty term of H,$\;J_{1}(H)=\left|\mathrm{sparseness}(H)-\mathrm{spH}\right|$, sparseness(H) is the sparsity of H after each update, and spH is an adjustable parameter ranging from 0 to 1. It can be adjusted according to the specific fluorescence signal distribution. By constraining the sparsity of H, it can help to eliminate the crosstalk between the decomposed images to obtain a purer fluorescence signal (Fig. 1cii).

### 2.4 Project gradient descent

Since the physical meaning of the feature matrix H can be regarded as the spectrum of each fluorophore, which has higher certainty compared with W, and the dimensionality of H is smaller than that of W, which is less computationally expensive and easier to find the optimal solution, gradient descent optimization of H is chosen. In this case, the update of H is transformed into a convex optimization problem, and W is obtained by multiplicative update. Since a sparsity constraint against H is added to the objective function, in order to guarantee the nonnegative limit, projected gradient descent (Hoyer 2003) is used here, and the algorithm essentially takes a step in the direction of negative gradients and then projects to the nonnegative constraint space (Fig. 1ciii). The objective function can be made to decrease at each step by ensuring that the step taken is small enough. The main power of the algorithm is the projection operator, which enforces the required sparsity and improves the repeatability and robustness of the algorithm. Algorithm 3 (Supplementary Note 3) shows the steps of projective gradient descent in BINGO.

### 2.5 Evaluation parameters

To evaluate the performance of BINGO, we borrowed some standard image evaluation parameters from other spectral unmixing works (Berry *et al.* 2007) and remote sensing hyperspectral unmixing to quantify and analyze the completeness of spectral unmixing. The ability of unmixing algorithms to eliminate image crosstalk can be obtained visually by calculating the two-dimensional correlation coefficient (2D-corr) (Qiao 2015, Bares *et al.* 2020) between different channel images and plotting the heat map (Fig. 1bi and bii). In addition, for experiments where the ground truth (GT) is known, we use the Structural Similarity (SSIM) (McRae *et al.* 2019, Su *et al.* 2019). Dice coefficient (Qiao 2015), which measures the image similarity, as the main evaluation index. For experiments where the ground truth is unknown, we calculate the root mean square error (Smith *et al.* 2020) (RMSE) and spectral angle distance (SAD) (Smith *et al.* 2020) to measure the completeness of the data after unmixing (Supplementary Note 4).

### 2.6 Sample preparation

Three-color BPAE (F36924, Invitrogen™) were with MitoTracker™ Red CMXRos labeling mitochondria, Alexa Fluor™ 488 phalloidin labeling F-actin, and DAPI labeling nucleus. 15-μm diameter polystyrene fluorescent beads (F8837 Blue, F8838 Blue-green, F21010 Green, F8844 Yellow-green, F21011 Yellow, F8841 Orange, F21012 Red-orange, F8842 Red, F8839, Crimson, F8843 Scarlet, Thermo Fisher, Scientific) were embedded in confocal imaging dishes with agarose. Take 10 μl of bead solution, centrifuge at low speed for 1 min, remove the supernatant and add an equal amount of other color beads solution, pipette and blow the solution to mix the beads thoroughly, centrifuge again, repeat the steps until all the beads are centrifuged, remove the supernatant, blow the beads with the residual liquid in the tube, take out all the suspensions and spread them in the middle wells of the confocal 24-well plate, and finally add 50/100 μl of 2% (wt/vol) agarose solution and wait for them to solidify.

For HeLa cells imaging, fusion proteins that linked different color fluorescent proteins to various intracellular proteins were expressed by transient transfection. We constructed four plasmids: pCAG-EBFP, pCAG-LSSmCherry-mClover3, pCAG-mKate- mCerulean, and pCAG-TagBFP-mAmetine to transfect in HeLa cells, respectively (Supplementary Table 1). All transfections were performed by using liposome (Lipofectamine™ 3000, Thermo Fisher) and incubating for eight hours. After another 12 hours, four dishes of transfected HeLa cells were mixed, and the fifth dish of HeLa cells incubated with CFSE (5 μM, 21888-25MG-F, Sigma) is added. The mixed cells were inoculated in confocal dishes and cultured in recommend medium supplemented (containing 89% DMEM medium, 10% FBS and 1% penicillin-streptomycin, Gibco) overnight (at 37°C in a 5% CO_2_ incubator). Cy3 (5 μM, ABS47038458, Absin) was used to stain the cell membrane before imaging.

### 2.7 Imaging

Two commercial microscopes were used in this work, including Nikon Ni-E and Zeiss LSM 780. The three-color BPAE for simulation experiments was acquired using Nikon Ni-E with Mai Tai HP Deepsee (690 nm-1040 nm) as the excitation source, and 750 nm was used. Three GaAsP NDD detectors with three filters (460/50,535/40, and 620/60) were used to collect fluorescence signal from DAPI, Alexa Fluor™ 488, and MitoTracker™ Red CMXRos, respectively. The acquisition was using Apo LWD 25 × 1.10 W DIC N2 Nikon objective. For spectral imaging of 10-color beads, Zeiss LSM 780 was used, which equipped with Mai Tai HP (690 nm-850nm) laser, W Plan-Apochromat 20×/1.0w objective, and 32-array GaAsP detector. The spectral imaging was set to 16 channels with a bandwidth of 17.9 nm per unit and a detection range of 410–695 nm. Nine--color HeLa cells were imaged by a two-photon microscope with exciton multiplexing. 25× objective (XLPLN25XWMP2, NA 1.05, 25×, Olympus) was used. A two-dimensional motorized precision stage (H117E1, Prior Scientific) was used for mosaic imaging. Signal was collected by photomultiplier tubes (H7422-40, Hamamatsu) through bandpass filters (FF02-447/60 or 475/50, FF01-525/45 or 520/60, FF01-629/53, Semrock, for blue, green, red fluorescence, respectively).

## 3 Results

### 3.1 Determination of spareness value

**Figure 2.**
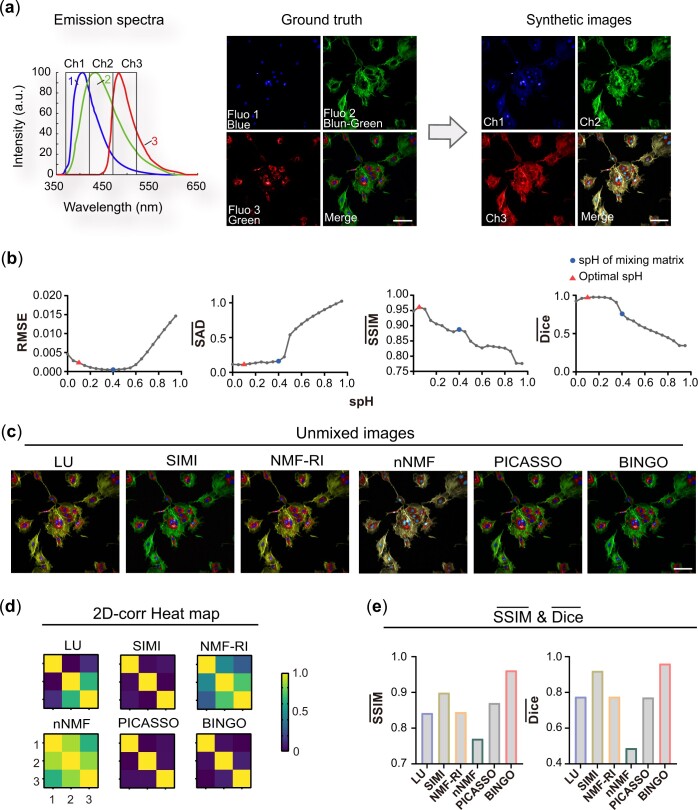
Determination of sparseness value and the unmixing performance through different algorithms. (a) Simulation of three-color BPAE images of fluorophores with highly overlapping emission spectra. (b) BINGO performance quantification while varying sparseness target value spH. 0.1 is the optimal value selected by considering all parameters (red triangle), which is slightly less than the sparseness of the mixing matrix (blue dot). (c) Unmixed composite and (d) 2D-corr heat map of different algorithms with spH = 0.1 (BINGO). (e) $\overset{-}{\mathrm{SSIM}}\;$and $\overset{-}{\mathrm{Dice}}$ values of different algorithms with spH = 0.1(BINGO). BINGO outperforms other algorithms. Scale bar: 100 μm.

We performed a unmixing simulation of synthetic three-color Bovine pulmonary artery epithelial cell (BPAE) images to demonstrate BINGO's superior ability to distinguish fluorophores from highly crosstalk images. Standard trichromatic BPAE images were used as the ground truth. Three fluorescent beads (Blue, Blue-green, and Green) with relatively similar spectra were selected for the simulation (Fig. 2a), minimal the distance between the wavelengths corresponding to the peak of the fluorescence spectrum was only 25 nm, and the overlap between them was as high as 0.78 (Supplementary Fig. S2a). The extractions of fluorophores can be achieved with a minimum of three 50-nm-wide detection channels, assuming a fluorescence collection range of 370 nm-520 nm and N_channel_/N_fluo_ = 1. A mixing matrix was generated based on the reference spectra and the corresponding channel settings. Then a multichannel simulation image was synthesized with ground truth (Fig. 2a, right), adding a moderate Gaussian noise and spectral shift (Supplementary Fig. S2b) to simulate the imaging scenario. The data set was input to various unmixing algorithms as the raw images. The mixing matrix was input as a reference spectrum for the LU, SIMI, and NMF-RI algorithms and as a strand for calculating the SAD. When inputting the original image into BINGO, we took the spH value from 0 to 1 to find an optimal value in BINGO with evaluation parameters.

The value of target sparseness spH of the feature matrix greatly impacts the results of BINGO (Fig. 2b). According to the definition of sparseness, the closer the sparseness is to 1, the more sparse the matrix H is. The narrower the width of the fluorescence spectrum and the larger the number of channels, the larger the sparsity of the feature matrix H should be. The value of spH should be set large enough to effectively reduce the crosstalk of the unmixed images. spH should be determined concerning the reference spectrum and the settings of the bandwidth and number of detectors. Figure 2b shows the performance of BINGO when the value of spH is changed from 0 to 1. In this simulation, the sparseness of the reference spectrum (mixing matrix) is 0.4. It can be seen that when spH > 0.4, RMSE, SAD, SSIM, and Dice all start to deteriorate, among which SAD and Dice are especially obvious, indicating that although the crosstalk between images is more minor with a larger spH value, it will lead to the optimization deviating from the optimal solution. When the spH is 0.4, the value of RMSE is the smallest, which means that the two matrices obtained from the factorization have the best descriptiveness of the image matrix, but the corresponding value of SSIM is smaller. In summary, spH should be set to be slightly less than the sparseness of the reference spectrum to obtain more satisfactory unmixing results, and the spH in all subsequent experiments was taken with this criterion.

We compared other algorithms and BINGO with optimal spH; the results are shown in Fig. 2c. The results show that BINGO obtains reliable unmixing results even without inputting reference spectra (Fig. 2c and Supplementary Fig. 2c). The performance of different spectral unmixing algorithms is quantified by 2D-corr heat map (Fig. 2d), SSIM and Dice coefficients of the similarity between the image and the ground truth, as shown in Fig. 2e, where$\;\overset{-}{\mathrm{SSIM}}\;$and $\overset{-}{\mathrm{Dice}}$ are averaged over the three fluorophores, which can quantify the overall performance of various algorithms in an experiment. The values of $\overset{-}{\mathrm{SSIM}}$ and$\;\overset{-}{\mathrm{Dice}}\;$for BINGO are the closest to 1 among all the algorithms, indicating that BINGO has a better separation ability for highly crosstalk fluorescence spectra when N_channel_/N_fluo_=1.

### 3.2 BINGO performance of fluorophores with dramatic intensity difference

**Figure 3.**
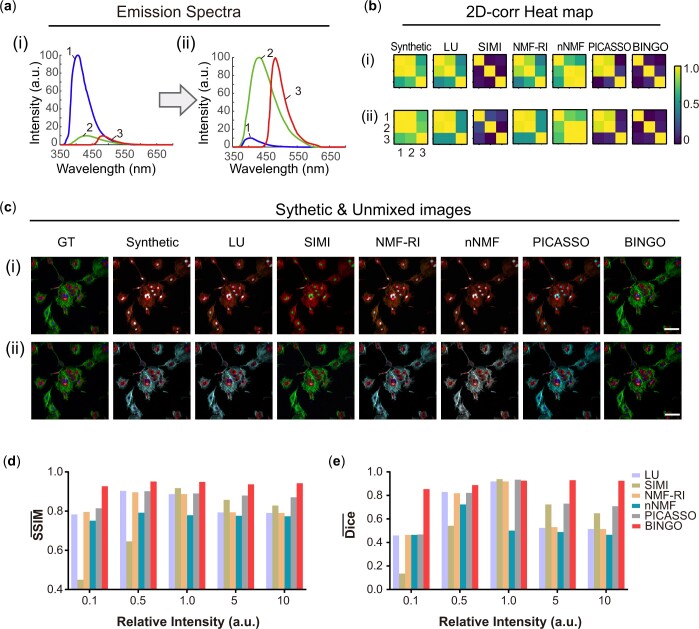
Unmixing performance of fluorophores with dramatic intensity difference. (a) Emission speactra of three-color BPAE simulation with dramatic intensity difference. (b) 2D-corr heat map and (c) composites of three Fluo 1, 2, and 3 after unmixing with different algorithms. The relative intensity of Fluo 1 and others is 10 (ai, bi, and ci) and 0.1 (aii, bii, and cii). (d) $\overset{-}{\mathrm{SSIM}}\;$and (e)$\overset{-}{\mathrm{Dice}}$ while changing the relative intensity of Fluo 1 and others from 0.1 to 10. $\overset{-}{\mathrm{SSIM}}\;$and $\overset{-}{\mathrm{Dice}}$ corresponding to BINGO are maintained at a larger value in all cases. Scale bar: 100 μm.

BINGO showed excellent performance in processing multichannel data with significant fluorescence intensity differences. For the three-color BPAE image mentioned in the previous section, we further consider the intensity difference between fluo1 and the other two fluorophores when constructing the mixing matrix. Assume that its fluorescence intensity is 0.1–10 times compared with the other fluorophores (Fig. 3a and Supplementary Tables S2 and S3), and then process them using different unmixing algorithms. Figure 3b and c shows the results solved by different algorithms when the relative intensity is 10 (Fig. 3bi and Supplementary Fig. S3a) and 0.1 (Fig. 3bii and Supplementary Fig. S3b), and it can be seen that only BINGO extracts all three fluorophores successfully at this time. The trends of $\overset{-}{\mathrm{SSIM}}$ and $\overset{-}{\mathrm{Dice}}$ values for different algorithms when the relative intensity increases are shown in Fig. 3d and e. The performance of other algorithms decreases sharply as the relative difference between fluorophores increases, and only BINGO keeps the value close to 1.

This simulation proved that BINGO works well even when fluorescence intensity varies greatly. In the typical spectral unmixing work, only fluorophores with comparable fluorescence intensity are considered. For fluorophores with significant differences in fluorescence intensity, images are reacquired after switching the excitation wavelength and it would cost more time to acquire the data. At this point, BINGO can extract the fluorophores with weaker intensity at single excitation can be extracted by spectral unmixing, the number of markers that can be used simultaneously in multicolor imaging can be further increased.

### 3.3 BINGO performance with a large number of fluorophores

**Figure 4.**
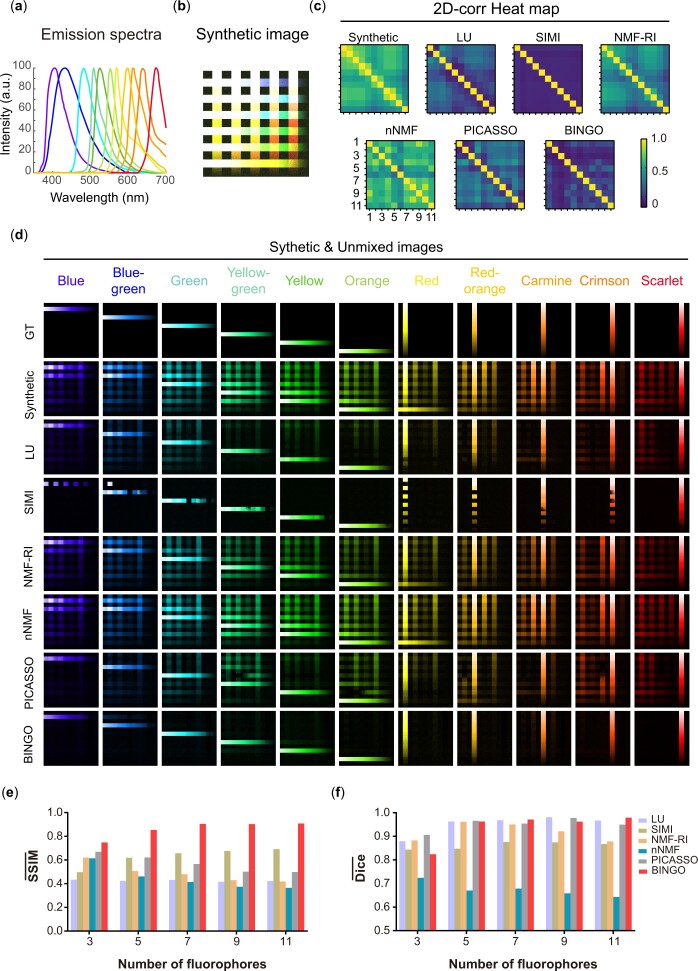
Unmixing performance while increasing the number of fluorophores. (a) Emission spectra and (b) synthetic composite while increasing the number of fluorophores to 11. (c) 2D-corr heat map and (d) individual images of 11 Fluo after unmixing with different algorithms. (e) $\overset{-}{\mathrm{SSIM}}\;$and (f) $\overset{-}{\mathrm{Dice}}$ while increasing the number of fluorophores from 3 to 11.$\;\overset{-}{\mathrm{SSIM}}\;$and $\overset{-}{\mathrm{Dice}}$ values corresponding to BINGO are maintained at a more considerable value while increasing the number of fluorophores.

To demonstrate the advantage of BINGO when dealing with a large number of fluorophores, we evaluated performance of different unmixing algorithms while increasing the number of fluorophores (Fig. 4a), and $\overset{-}{\mathrm{SSIM}}$ and $\overset{-}{\mathrm{Dice}}$ values were still calculated (Supplementary Tables 4 and 5). The experiment used strips to simulate different fluorophores to generate a grayscale image as the ground truth (Fig. 4b, first row) and kept N_channel_/N_fluo_=1. Then it added Gaussian noise and spectral shift (Supplementary Fig. S4a and b) to simulate the actual imaging conditions after calculating the mixing matrix based on the reference spectrum and channel width to generate the synthetic images (Fig. 4b, second row and Supplementary Fig. 4d).

The fluorophore spectra used in the experiments were referenced to all types of commercially available fluorescent beads (Fig. 4a), Blue, Blue-green, Green, Yellow-green, Yellow, Orange, Red-orange, Red, Carmine, Crimson, and Scarlet) with emission spectra in the visible range, each of which has a similar fluorescent protein or fluorescent dye that can be used for bio-specific labeling. Considering the visible detection range, the width of the fluorophore, and the conventional width of the Stokes shift, the relative intensity between fluorophores, and other practical factors, eleven fluorophores are beyond the current limit of the number of multicolor schemes required for a single excitation. Complex ultra-multicolor labeling scenarios with as many as eleven targets in biological systems can be simulated.

Figure 4c and d shows the simulation results obtained by different algorithms for multichannel images when the number of fluorophores is increased to 11. As can be seen from the images, except for BINGO and SIMI, all other algorithms fail to extract each fluorophore and fail to completely eliminate image crosstalk. However, SIMI fails to deal with the spatial overlap of fluorophores because it uses the classification idea for unmixing, resulting in low values of $\overset{-}{\mathrm{SSIM}}$ and $\overset{-}{\mathrm{Dice}}.$

From the comprehensive data in Fig. 4e and f, BINGO also shows a considerable advantage over other algorithms. As the number of fluorophores grows, the image solved by BINGO maintains a high similarity to the ground truth (both $\overset{-}{\mathrm{SSIM}}$ and $\overset{-}{\mathrm{Dice}}\;$remain at a high level). The simulation shows that the advantage of BINGO when N_channel_/N_fluo_=1is more apparent with the large number of fluorophores. Compared to random initialization, NNDSVD helps the optimization process saved half the time (Supplementary Fig. S5).

### 3.4 Experimental fluorescent beads images unmixing

We then performed spectral imaging using the standard fluorescent beads mentioned in the previous section. Since the actual color of each fluorescent bead is relatively easy to distinguish under the naked eye, the fluorescent beads in the images can be manually classified to obtain the ground truth of each fluorophore, which is the standard image in the subsequent evaluation (Supplementary Fig. S6a and c).

**Figure 5.**
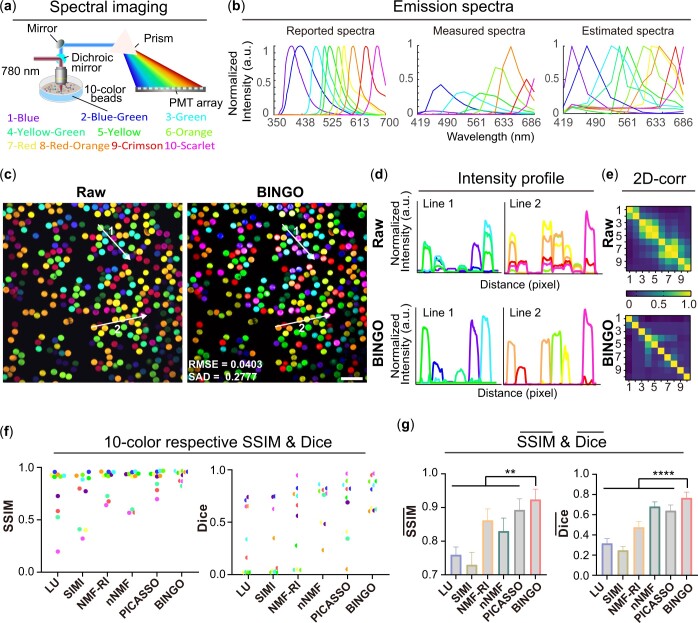
BINGO with experimental 10-color fluorescent beads. (a) 15-channel spectral imaging with ten-color beads. (b) Reported, measured, and estimated spectra by BINGO of 10 fluorescence beads. Colors of the different lines in the plot correspond to the fluorophores’ names in Figure 6a. (c) BINGO results of experimental data from 10-color fluorescence beads show more distinctive(right) than raw data(left). (d) Intensity profiles of each bead for raw images(top) and BINGO results(bottom). (e) 2D-corr heat map of raw images(top) and BINGO results(bottom). (f) Respective SSIM and Dice scattering plot of each bead. (g) Mean and square difference of $\overset{-}{\mathrm{SSIM}}\;$and $\overset{-}{\mathrm{Dice}}$ across 25 data sets. Colors in (b-d, f) match rendering in (a) and numbers in (e) correspond to fluorescent beads in (a). Scale bar: 50 μm.

The two-photon excitation spectrum is broader than the single-photon, so more fluorescent beads can be excited with single excitation. We used a femtosecond laser with a wavelength of 780 nm as the light source to simultaneously excite 10 fluorescent beads (1—Blue, 2—Blue-green, 3—Green, 4—Yellow-green, 5—Yellow, 6—Orange, 7—Red-orange, 8—Red, 9—Crimson, and 10—Scarlet). The unit bandwidth was set to 17.8 nm, and 15 channels of spectral imaging (Fig. 5a) could be performed. In Fig. 5b, the reported spectra represent the fluorescence spectra provided by the manufacturer. The measured spectra are the fluorescence intensity distribution of fluorophores in each channel in the experimental spectral imaging, normalized to the grayscale value in the channel with the strongest fluorescence intensity under the same excitation conditions. Comparing the reported spectra with the measured spectra, the spectra of fluorescence beads in the actual imaging are very different from the reported spectra, regardless of the wavelength of the peak or the full width at half maximum (FWHM). Measuring the spectra in advance each time makes the experimental steps more cumbersome, and for intravital imaging it is difficult to measure absolutely accurate spectra in advance because the complex biomedical environment significantly perturbs the spectra. So in these cases, the use of a reference-based unmixing method will introduce a large error. In addition, there is a significant difference in fluorescence intensity between different fluorescence beads, and the fluorescence intensity of Blue and Yellow-green fluorescence beads is nearly 10 times different from the brightest Red-orange ratio at this time due to the long distance between the excitation source and its optimal excitation wavelength, which does not meet the conditions for PICASSO use. And the spectra of 4—Yellow-green and 5—Yellow were extremely similar, and the peak gap and spectra overlap of them were about 16 nm and 0.75, respectively.

In Fig. 5b and Supplementary Fig. S7, the estimated spectra are the spectra calculated by BINGO. The wavelength of the peak and width of each fluorophore spectrum is similar to the measured one, which reflects the accuracy of the estimated spectra by BINGO. From measure spectra, we can see that the fluorescence intensity of yellow-green fluorescent beads and their neighboring green and yellow fluorescent beads are significantly different, which makes their extraction difficult, so after the algorithm outputs the unmixed images, another image subtraction is performed. To further separate the fluorophores, we subtract the green and yellow counterparts from the output yellow-green image (all algorithms perform this operation). Figure 5c shows the fluorescent beads before and after BINGO, and Fig. 5d shows the intensity profiles of the two lines in Fig. 5c for different fluorescent beads. The fluorescent beads are tiled in a single layer, so there should be no spatial overlap in the image, but due to signal crosstalk, different color curves of raw images overlap spatially in a very obvious way, while after unmixing using the BINGO, the different color curves hardly overlap anymore. As seen from the 2D-corr heat map before and after BINGO in Fig. 5e, the crosstalk between images is eliminated.

We found that the extraction completeness of different fluorophores by the same algorithm varies depending on the overlap of the fluorophore spectra and the relative fluorescence intensities. Fluorophores with more vigorous fluorescence intensity and narrower fluorescence spectra tend to be extracted more purely, while the opposite extraction is more complicated. SSIM and Dice were calculated from each of the fluorophores obtained by different algorithms (Fig. 5f and Supplementary Tables S6 and S7). It is found that the scatter points of the other algorithms used in the paper, both SSIM and Dice of Blue, Yellow-green, and Scarlet, tend to be distributed below the mean value due to the weak relative fluorescence intensity, with the difference of Dice values being more noticeable. The difference between Blue and Yellow-green, corresponding to the ground truth, can also be seen in the single-channel images (Supplementary Fig. S6c). However, these indexes of BINGO to all fluorophores corresponding to scatter points on the scatter plot are concentrated at positions with larger values, which shows that BINGO can extract all fluorophores more accurately than other algorithms, regardless of the severe crosstalk of the fluorescence spectrum or the dramatic intensity differences between fluorophores. In addition, to check the repeatability and robustness of the BINGO, we counted the expectation and variance (Fig. 5g) of the experimental data (*N* = 25) corresponding to $\overset{-}{\mathrm{SSIM}}$ and $\overset{-}{\mathrm{Dice}}$. BINGO demonstrated stronger fluorophore distinction and robustness with the largest value of $\overset{-}{\mathrm{SSIM}}$ and $\overset{-}{\mathrm{Dice}}$, indicating the highest similarity to the standard image and demonstrating a significant difference (*P* < .0001). With the results of experimental fluorescent beads images, BINGO shows good performance even if the spectra of the fluorophores are extremely similar or their fluorescence intensity varies greatly.

### 3.5 Nine-color HeLa cells unmixing

**Figure 6.**
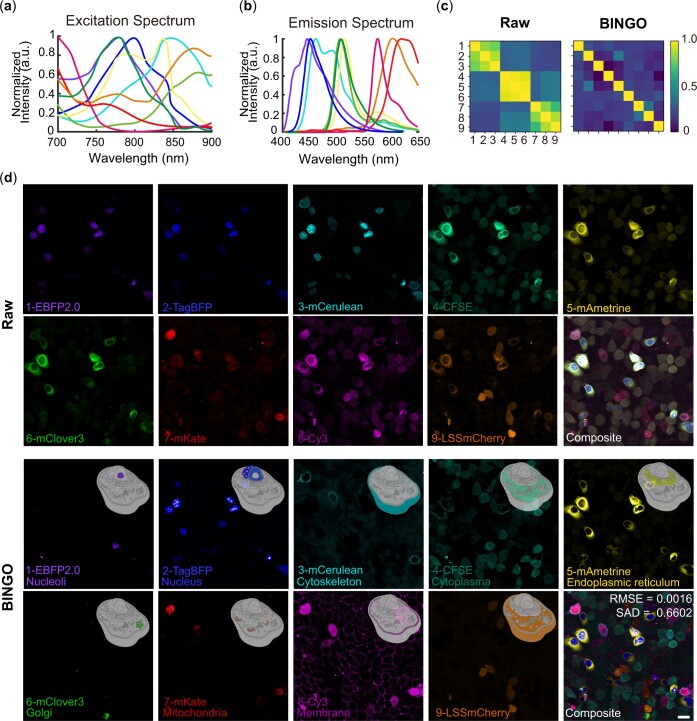
BINGO with experimental nine-color HeLa cells. (a) Two-photon excitation spectra and (b) emission spectra of nine fluorophores labeling HeLa cells. (c) 2D-corr heat map of raw images and BINGO results. (d) BINGO results of experimental data from 9-color HeLa cells show more distinctive(bottom) than raw data(upper). Colors in (a, b) match rendering in (d) and numbers in (c) correspond to fluorescent beads in (d). Scale bar: 20 μm.

Further, nine-color HeLa cells with a two-photon excitation multiplexed multichannel detection microscope were successfully distinguished after BINGO. We selected nine fluorophores, including seven fluorescent proteins and two fluorescent dyes. Their two-photon excitation spectra and emission spectra are shown in Fig. 6a and b. The fluorescent protein EBFP2.0 was fused to fibronectin so that EBFP2.0 was expressed in the nucleolus; TagBFP was fused to H2B and expressed throughout the nucleus; mCerulean was fused to Lifeact and expressed in the cytoskeleton; mAmetrine was fused to Calreticulin and KDEL and expressed in the endoplasmic reticulum. mClover3 is fused to SiT-15 and expressed in the Golgi; mKate is fused to pyruvate dehydrogenase and expressed in the mitochondria; and LSSmCherry is expressed in the whole cell. In addition, CFSE and Cy3 were stained directly by incubation, with CFSE covalently bound to the cytoplasm and Cy3 bound to the phospholipid bilayer on the cell membrane (Fig. 6d).

Because of the broad two-photon absorption spectrum, the crosstalk during excitation multiplexing is more serious, as seen in the 2D-corr heat map of raw images in Fig. 6c. in this situation, N_channel/_N_fluo_=1, the assumption of “unidirectional crosstalk” is no longer available, and none of the raw images contains a single pure fluorophore, unmixing was more difficult, algorithm mentioned above all cannot work well. Even then, BINGO removed the crosstalk significantly in this extremely complex situation, the unmixing results in Fig. 6d show that the subcellular localization of each fluorophore is in perfect agreement with the previously designed labeling strategy. Even small Golgi apparatus, tiny fibrils composed of cytoskeleton, or Cy3-labeled cell membranes and nucleoli with relatively weak signals can be accurately extracted from the raw images. Thanks to TagBFP and mAmetrine were linked in a plasmid; mCerulean and mKate were linked in a plasmid; LSSmCherry and mClover3 were linked in a plasmid, so these pairs of proteins would be expressed in the same cell. BINGO can differentiate between different fluorescent proteins when they are expressed in the same cell and BINGO’s ability to extract fluorophores for live cell imaging was verified.

## 4 Conclusion

We present here a multichannel image processing method-BINGO, which can assist in imaging multiple highly complex overlapping fluorescent labels simultaneously without any reference spectra. Through simulation, BINGO can separate 11 fluorophore images in single excitation at N_channel_/N_fluo_=1 with spectral overlap up to 0.78, and the number of the fluorophore, the extraction purity, and accuracy are significantly higher than other algorithms. For experimental data, BINGO achieves accurate extraction of 10 fluorophores excited by a single excitation source, which is the most number of fluorophores that can be distinguished in a single round of excitation in fluorescence imaging and 9-color HeLa cells were successfully distinguished by BINGO. Compared with other existing algorithms, BINGO shows excellent performance in dealing with fluorophores with greater differences in fluorescence intensity, achieving successful extraction for all fluorophores in the sample, obtaining purer images, and achieving more accurate quantitative analysis results.

Blind source separation is a classical problem in signal processing, which refers to separating each source signal from a mixed signal (observed signal) when the theoretical model of the signal and the source signal are not precisely known. Principal component analysis based on SVD, k-means clustering analysis (McRae *et al.* 2019, Bares *et al.* 2020), etc. draw on dimensionality reduction algorithms, which apply to the scenario that the channel number is more over than fluorophores. Moreover, support vector machine SVM (Rehman and Qureshi 2021), autoencoder (Su *et al.* 2019), and convolutional neural network (Smith *et al.* 2020) can also perform blind source spectral unmixing. However, these deep learning methods need a large number of data sets for training and have a higher barrier to use. BINGO, as a blind unmixing algorithm, results do not depend on acquiring a priori fluorescence spectra, eliminating the need for a time-consuming, complex, and sometimes impossible reference measurement process. So, BINGO is therefore very suitable for intravital imaging.

BINGO is less restrictive on the input images and tolerates higher crosstalk between input images, which minimizes the number of detection channels required and allows a larger channel bandwidth for spectral imaging, maximizing the number of photons collected per channel and ensuring a higher SNR. No “unidirectional crosstalk” is required, which is friendly for excitation multiplexing of multichannel data or fluorophores with dramatic intensity difference. Whether multichannel acquisition with bandpass filters or spectral imaging systems, BINGO can handle them all and is highly system compatible with most commercial fluorescence microscopes and new optical systems in the laboratory today. A number of algorithms have also emerged to distinguish observables based on target morphology (Megjhani *et al.* 2017, Maric *et al.* 2021), which, in combination with BINGO, have the potential to further increase the number of simultaneous targets.

BINGO shows good robustness when processing more than 10 fluorophores, enabling high-content intravital imaging. BINGO as a multichannel image processing tool is potential to be used for high-throughput molecules and proteins analysis with fluorescence intensity, spectra, or lifetime. For complex cellular dynamics (Liang *et al.* 2022), tumor microen-vironment (Lee *et al.* 2021), or chemical compositions analysis (Gleeson *et al.* 2023), BINGO can provide a new tool to achieve multi targets spatial analysis. BINGO breaks through the intrinsic problem of fluorescence spectral crosstalk, allowing higher quality extraction of highly crosstalk fluorophores, relaxing the number limitation of simultaneous observation of multiple targets, and assisting mechanism research in biomedicine as an algorithmic tool.

## Supplementary Material

btae052_Supplementary_Data

## Data Availability

The data underlying this article will be shared on reasonable request to the corresponding author.
