# Supplementary material for: BINGO: a blind unmixing algorithm for ultra-multiplexing fluorescence images
Source: Bioinformatics. 2024 Jan 30;40(2):btae052. doi: 10.1093/bioinformatics/btae052 (PMC10873573; doi:10.1093/bioinformatics/btae052)
Supplement: btae052_Supplementary_Data [file btae052_supplementary_data.pdf]

BINGO: a blind unmixing algorithm for ultra-multiplexing fluorescence images

List of content

- **Supplementary Notes**
- **Supplementary Figures**
- **Supplementary Tables**

Supplementary Note 1 The generic NMF Unmixing

**Algorithm 1 ： The generic NMF Unmixing**
**Input**：*X, r,* ε
**Output**：*W* and *H*
**Initialization**：*W0* and *H0*
**Set:** Objective Function: $F = \|X - WH\|$
1 Checking Stop Condition(s)
2 **If** ， $\|x^k - x^{k-1}\| < \varepsilon$， **then**
3 finish algorithm
4 **Else**
5 trial step calculation：Update W and H

According to linear mixing model of spectra recognized by fluorescence imaging over the years (Zimmermann 2005, Zimmermann *et al.* 2014, Rakhymzhan *et al.* 2017), the total detected fluorescence signal for every channel λ can be written as $\mathrm{I}(\lambda)$,

$$\mathrm{I}(\lambda) = A_1 Fluo_1(\lambda) + A_2 Fluo_2(\lambda) + \cdots + A_r Fluo_r(\lambda). \quad (1)$$

$Fluo_x(\lambda)$ represents the spectral contribution of the fluorophores to every channel, and A_x represents the abundances of the fluorophores in the measured spot.

On the other hand, BINGO was designed with NMF, the decomposition structure of NMF is

$$X = WH\ . \quad (2)$$

where, $\mathrm{X} = [\mathrm{I}(\lambda_1), \mathrm{I}(\lambda_2), \cdots, \mathrm{I}(\lambda_n)]$, X is a flatten multi-channel image matrix with a scale of $m \times n$, *m* denotes pixel number of each image and *n* denotes number of channels. W and H are called the abundance and feature matrices, $W \in R^{m\times r} and\ H \in R^{r\times n}$, respectively. What *r* represents is the number of fluorophores in the sample, $r \le \min{(m, n)}$. The abundance matrix W, as the name suggests, $\mathrm{W} = [\mathrm{A}_1, \mathrm{A}_2, \cdots, \mathrm{A}_r]$, represents the spatial concentration distribution of each fluorophore, and the corresponding image of each fluorophore is obtained after rearranging the matrix to an image; $\mathrm{H} = [Fluo_1, Fluo_2, Fluo_r]^T$, the feature matrix H reflects the intensity distribution of each fluorophore in each channel, corresponding to its estimated spectrum.

$$[\mathrm{I}(\lambda_1), \mathrm{I}(\lambda_2), \cdots, \mathrm{I}(\lambda_n)] = [\mathrm{A}_1, \mathrm{A}_2, \cdots, \mathrm{A}_r] \times \begin{bmatrix} Fluo_1 \\ Fluo_2 \\ \vdots \\ Fluo_r \end{bmatrix} \quad (3)$$

Supplementary Note 2 NNDSVD Initialization

Algorithm 2 shows the steps of NNDSVD Initialization. Input the image matrix X and the number of fluorophores to be solved, r, to output the initialization value *W0* and *H0.* At first, it performs a singular value decomposition of the image matrix X with r as the rank, and the goal of the second SVD is to calibrate all elements of the matrix to non-negative, which mainly based on equation (4).

$$X = \sigma_1 u_1 {v_1}^T + \sigma_2 u_2 {v_2}^T + \cdots + \sigma_r u_r {v_r}^T \quad (4)$$

The principle of the second SVD is as follows. The elements in u_j and v_j include both positive and negative elements. u+ is written as all the elements of ≥ 0 in vector u, and the rest are replaced by 0. u- is written as all the elements of < 0 in vector u, and the rest are replaced by 0. Similarly for vector v, at this point,

$$\begin{aligned} C = u_j {v_j}^T &= (u_+ - u_-)(v_+ - v_-)^T \\ &= (u_+ {v_+}^T + u_- {v_-}^T) - (u_+ {v_-}^T + u_- {v_+}^T) \\ &= C_+ - C_- \approx C_+ \end{aligned} \quad (5)$$

After ignoring C_-, all elements less than 0 can be corrected. $\hat{u}_\pm := u_\pm / \|u_\pm\|$, $\hat{v}_\pm := v_\pm / \|v_\pm\|$, $\mu_\pm = \|u_\pm\|\|v_\pm\|$, here $[\hat{u}_+, \hat{u}_-]$ and $[\hat{v}_+, \hat{v}_-]$ became unitary matrix. $\mu_\pm$ were singular value. writing C_+ with equation (4),

$$C_+ = \mu_+ \hat{u}_+ {\hat{v}_+}^T + \mu_- \hat{u}_- {\hat{v}_-}^T \quad (6)$$

Since rank（C）=1, rank（C_+）is at most 2, we can approximate C_+ by the larger term on the right-hand side of Eq. (6). The initial non-negative matrices *W0* and *H0* are obtained after a low-rank approximation for each of the latter terms. Although NNDSVD gives up part of the similarity in the process of calibrating negative elements, the features extracted by SVD are retained to the maximum extent, which can be compensated in the subsequent iterative optimization.

Algorithm 2 ： NNDSVD Initialization

Input： *X, r*

Output： *W0* and *H0*

1 Compute the largest r singular triplets of X: $X = U\Sigma V^T$

2 **for** $j = 2{:}r$

3 $u = U(:,j); v = V(:,j)$

4 $u_+ = pos(u); u_- = neg(u); v_+ = pos(v); v_- = neg(v);$

5 **if** $\|u_+\|\|v_+\| > \|u_-\|\|v_-\|$, $\mathrm{W0}(:,\mathrm{j}) = \sqrt{\Sigma(\mathrm{j},\mathrm{j}) \times \|u_+\|\|v_+\|} \times u_+/\|u_+\|$

$\mathrm{H0}(j,:) = \sqrt{\Sigma(\mathrm{j},\mathrm{j}) \times \|u_+\|\|v_+\|} \times {v_+}^T/\|v_+\|$

6 **else** $\mathrm{W0}(:,\mathrm{j}) = \sqrt{\Sigma(\mathrm{j},\mathrm{j}) \times \|u_-\|\|v_-\|} \times u_+/\|u_+\|$

$\mathrm{H0}(j,:) = \sqrt{\Sigma(\mathrm{j},\mathrm{j}) \times \|u_-\|\|v_-\|} \times {v_-}^T/\|v_-\|$

end

Supplementary Note 3 Project gradient descent

Algorithm 3 ： Project gradient descent

Input： W^k *and* H^k ,μ

Output： W^{k+1} and H^{k+1}

1 **Set:** $H \coloneqq H - \mu W^T(WH - V)$

2 Project each row of H to be non-negative, have unit L_2 norm, and L_1 norm set to achieve desired sparseness

3 $W \coloneqq W * (VH^T)/(WHH^T)$

Supplementary Note 4 Evaluation parameters

4.1 RMSE

RMSE is the most commonly used index to assess the completeness of data after spectral unmixing without additional spectral information. The smaller the RMSE value, the less information is lost.The RMSE is calculated as

$$RMSE = \sqrt{\frac{1}{p \times m} \|V - WH\|^2} \tag{7}$$

where p is the number of pixels in each image, m is the number of image channels, and this reference value is proposed in the hyperspectral decomposition to be less than 0.1.

4.2 SAD

For blind source separation (BSS), the extracted spectrum can be compared with the actual measured spectrum and the SAD (spectral angular distance) is calculated to assess the similarity of the two spectra. The larger the difference between the two spectra, the larger the corresponding spectral angular distance value, and the smaller the value indicates that the two spectra are more similar. For two vectors a and b,

$$SAD(\boldsymbol{a}, \boldsymbol{b}) = \arccos\left(\frac{a^T b}{\|a\|_2 \|b\|_2}\right) \tag{8}$$

For each fluorophore, a SAD value is calculated, and the average value of all fluorophores $\overline{\text{SAD}}$ is often used to measure the overall capability of the spectral unmixing algorithm during a single experiment.

4.3 Correlation coefficient

The 2D correlation coefficient is usually used to examine the similarity between two images, which can be calculated by the corrcofe function in MATLAB. The two-dimensional correlation numbers between different channel images are plotted as a heat map (Fig1b). The horizontal and vertical coordinates of the heat map all correspond to fluorophore types, and are represented by 1, 2, 3, ..., which can visualize the crosstalk between images. the 2D correlation between each channel image and itself is 1, and the value of 2D correlations with other channels should be close to 0. Calculating the 2D correlation coefficients between different channel images can quantify the degree of crosstalk between all channel images.

4.4 SSIM

For data that can provide ground truth, the SSIM (Structural Similarity) between the unmixing result and the standard image can be calculated, which can be calculated by the ssim function in MATLAB. SSIM is a widely used image quality evaluation indicator that evaluates the similarity between two images based on the assumption that the human eye extracts structured information from them when viewing an image. As with SAD, an SSIM value is calculated for each fluorophore, and the average value of all

fluorophores $\overline{SSIM}$ is generally used to measure the overall capability of the spectral unmixing algorithm. the SSIM takes a value between [0,1], and the closer to 1 means the more similar the image is, the more accurate the unmixing result is.

4.5 Dice

After binarizing the fluorophore grayscale image, structural information such as area and volume of the target can be obtained by counting the number of pixels. The Dice coefficient is an ensemble similarity measure function that is usually used to calculate the similarity of two samples and takes the value of [0,1]. Unlike SSIM, which can measure similarity for grayscale images, the Dice coefficient can only be used for binarized images. If the Dice coefficient between the unmixing result after binarization and the standard image is large, it means that the result of the later image analysis is closer to the true value and the result is more reliable.

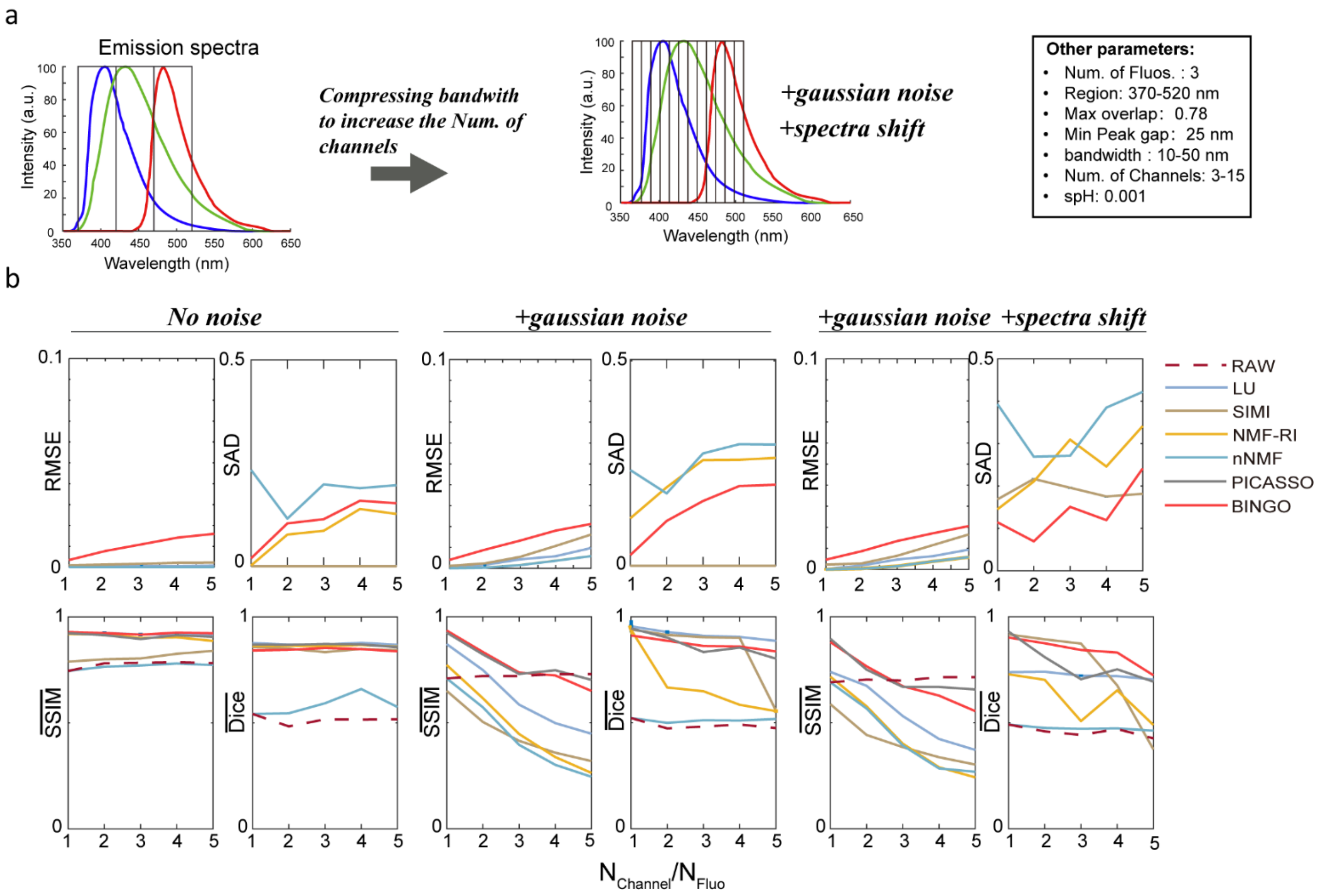

Supplementary Fig. 1│ Unmixing performance while compressing the bandwidth of channels. a Grapical represent of Simulation that compressing band width to increase the number of channels from three (50 nm bandwidth) to fifteen (10 nm bandwidth) with three fluorophores. To simulate the noise and spectral shifts in the imaging experiments, we performed three simulations of no noise, gaussian noise, and both gaussian noise and spectra shift respectively.**b** RMSE, SAD, SSIM, and Dice of different algorithms in the simulation. Three panels from left to right denotes results in the situations of with no noise, with gaussian noise and with both gaussian noise and spectral shift. Because noise is unavoidable in the experiment, compressing the channel width would deteriorate performance of all unmixing, and a wider band width in spectral imaging is more conducive to acquiring accurate and high-quality images. when there were spectral shifts in the speciman, the results obtained by the reference-based algorithm (LU, SIMI, and NMF-RI) become worse, and the blind unmixing (PICASSO, and BINGO) obtains more reliable results.

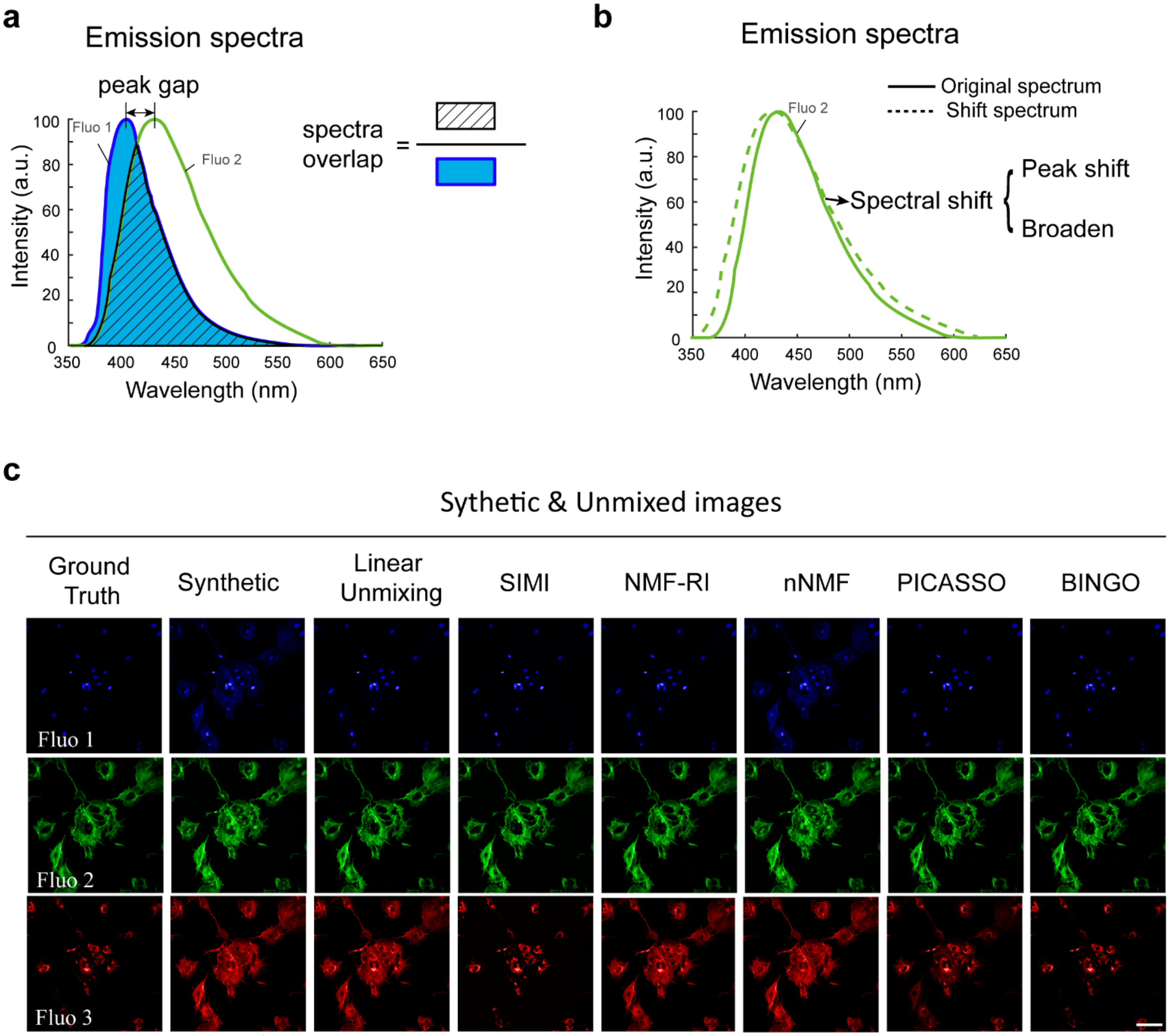

Supplementary Fig. 2│ Measurement of the crosstalk degree between two fluorophores and Single-channel image of Fig.2. a Evaluation criterion of overlap between two fluorophores' emission spectra in

the paper, take Fluo 1(blue solid line) and Fluo 2(green solid line) for example, both spectra have been normalized itself respectively. Peak gap means the distance between the wavelengths corresponding to the their emission peak. Spectral overlap means the ratio between the area of the overlapping region(diagonal stripe region) and the area of the spectrum itself(blue region).**b** Emission spectrum of Fluo 2 in Fig. 2 and Fig. 3 before(solid line) and after(dotted line) spectral shift.Simulation included peak shift and boarden, the corresponding wavelength of the peak shifted to shorter wavelength about 5 nm, and the FWHM of it boarden to about 1.2 times. **c** Single-channel image of ground truth, sythetic images, and the results of different algorithms. Scale bar: 100 μm.

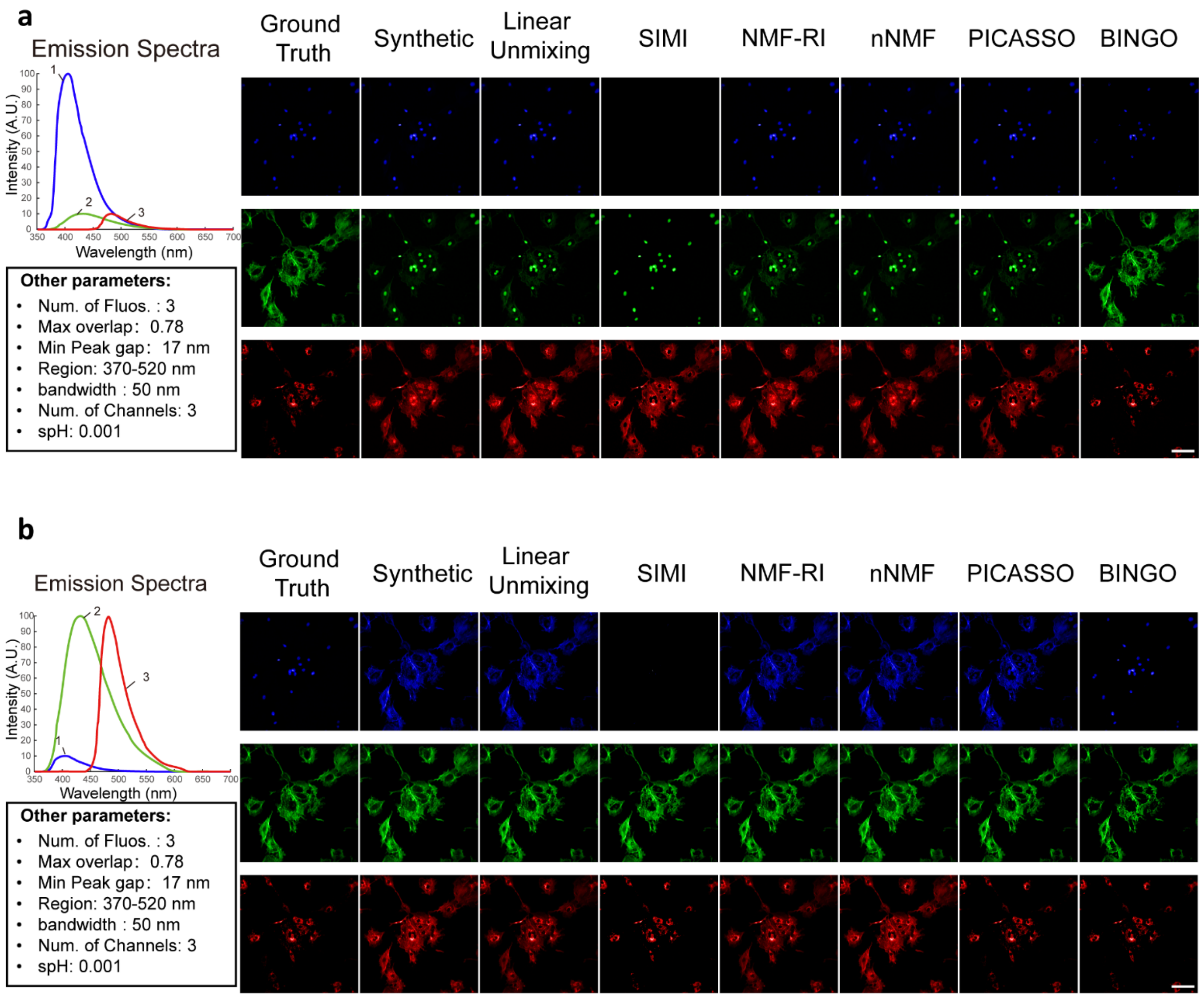

Supplementary Fig. 3 | Unmixed images of fluorophores with dramatic intensity difference. a Single-channel images of ground truth, sythetic images, and the results of different algorithms in Fig.3bi. Relative intensity of Fluo1 and others was 10, other parameters were listed in black box in detail. **b** Single-channel images of ground truth, sythetic images, and the results of different algorithms in Fig.3bii. Relative intensity of Fluo1 and others was 0.1, other parameters were listed in black box in detail. Scale bar: 100 μm.

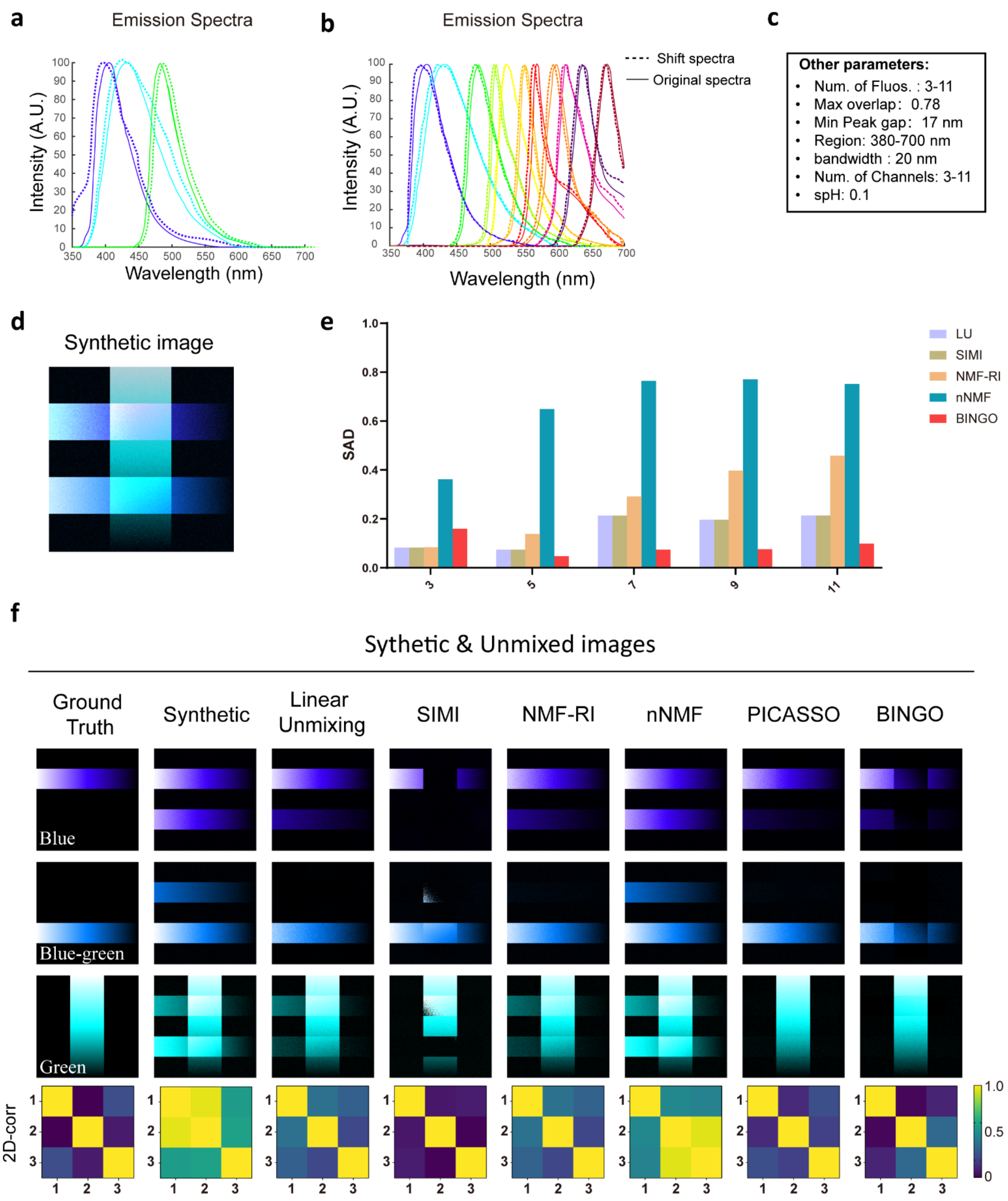

Supplementary Fig.4 | Unmixing performance while increasing the number of fluorophores. a Original (solid lines) and shifted (dotted lines) spectra of three fluorophores in Fig.3ai **b** Original (solid lines) and shifted (dotted lines) spectra of eleven fluorophores in Fig.3aii. The peak shift of each

fluorophore were all set about 0~ 8 nm to shorter or longer wavelength randomly, and broaden about 1~1.5 times randomly. **c** Detailed parameters used in Fig.3. **d** Synthetic image of 3 color image. **e** $\overline{\text{SAD}}$ value while increasing the number of fluorophores from 3 to 11. BINGO achieved the smaller $\overline{\text{SAD}}$ value compared to the other algorithms, indicating that its estimated spectra are closer to those after the artificial spectral shift. **f** Single-channel images and 2D-corr heat map of three fluorophores in Fig.3ai after unmixing with different algorithms.

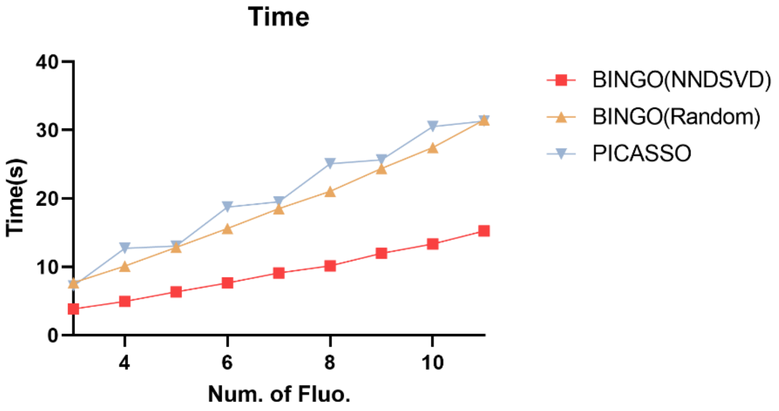

Supplementary Fig. 5 | Speed comparison of multiple blind unmixing algorithms. The time consumed by the blind unmixing algorithm in Fig. 4. Regardless of the bling unmixing algorithm, the computation time increases as the number of fluorophores increases. With NNDSVD initialization in BINGO, the calculation time is halved compared to both random initialization of BINGO and PICASSO.

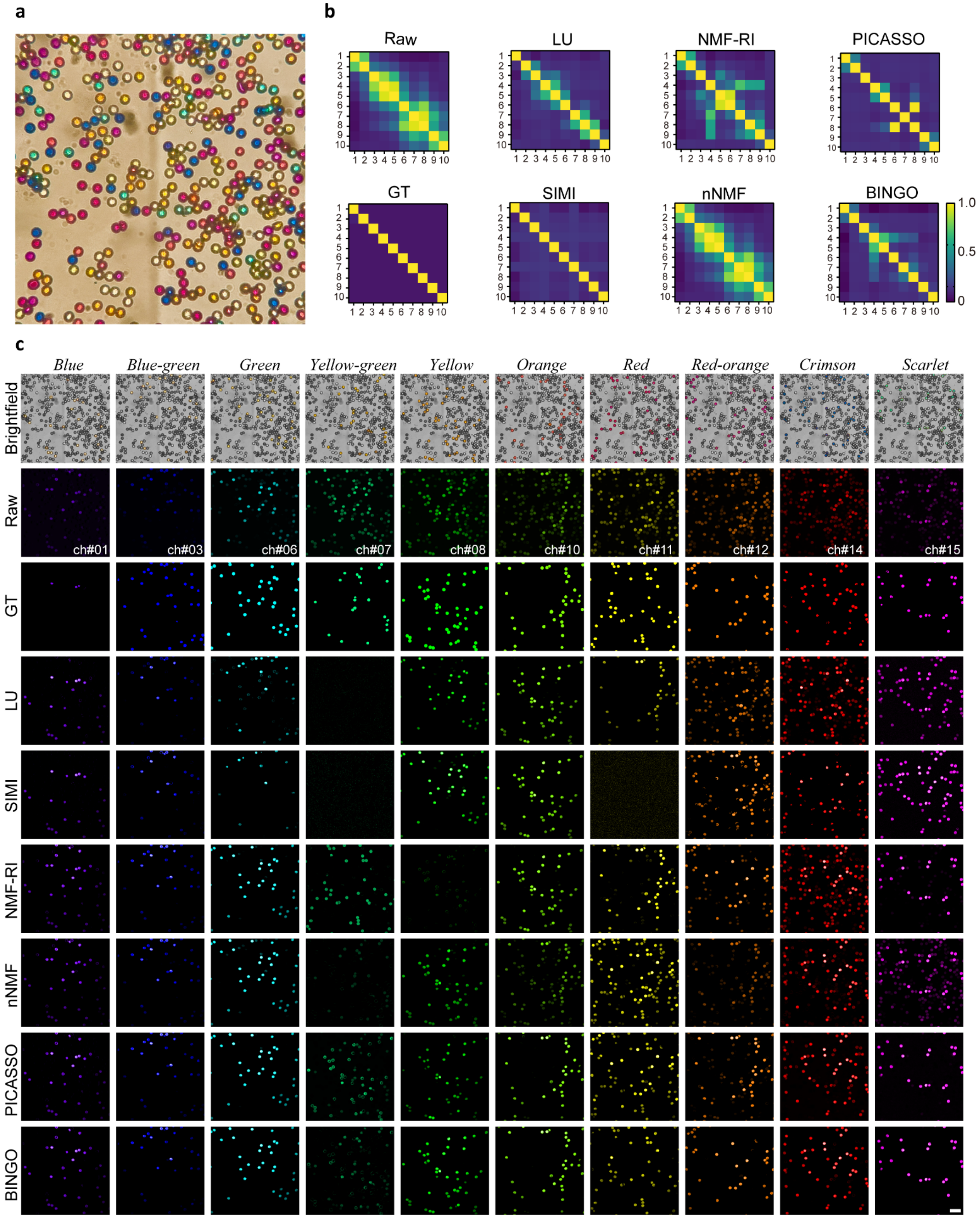

a
b
Raw
LU
NMF-RI
PICASSO
GT
SIMI
nNMF
BINGO
1.0
0.5
0
c
Blue
Blue-green
Green
Yellow-green
Yellow
Orange
Red
Red-orange
Crimson
Scarlet
Brightfield
Raw
GT
LU
SIMI
NMF-RI
nNMF
PICASSO
BINGO
ch#01
ch#03
ch#06
ch#07
ch#08
ch#10
ch#11
ch#12
ch#14
ch#15

Supplementary Fig. 6｜Unmixed results of experimental ten-color fluorescence beads. a Photo of 10-color fluorescent beads under bright field. **b** 2D-corr heatmap of ground truth, raw images, and the results of different algorithms. **c** Single-channel image of ground truth, raw images, and the results of different algorithms. Scale bar: 50 μm.

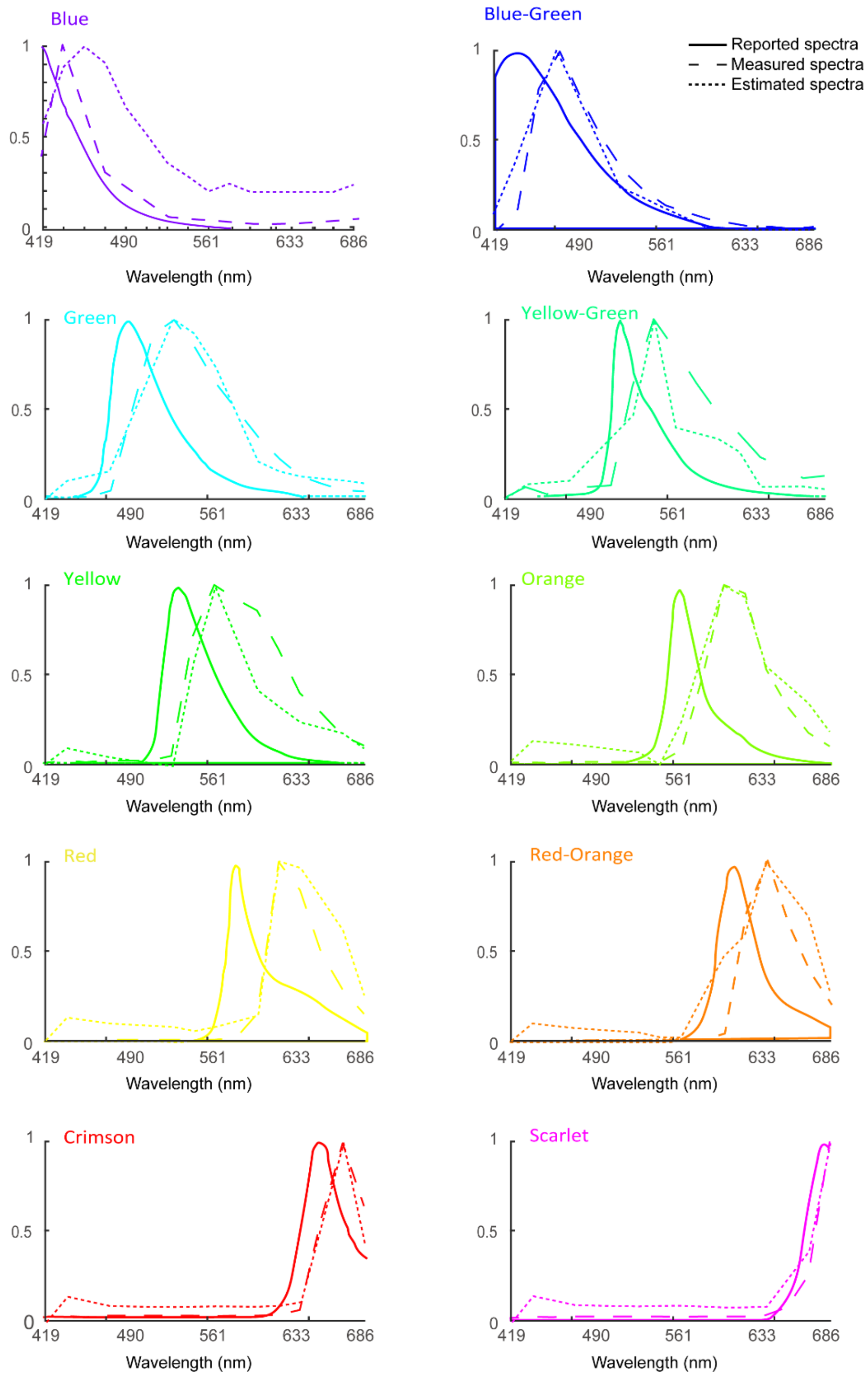

Supplementary Fig.7│ Comparison of emission spectra for experimental ten-color fluorescence beads in Fig.5. For each of the ten bead colors used in this work, we show the reported emission spectrum(dotted lines) from the manufacturer(Thermo Fisher), the measured emission spectrum(dotted lines) with spectral imaging with Nikon LSM780, and the estimated emission spectrum(solid lines) with BINGO. Comparing reported spectra with measured spectra, most of these dyes shift to longer wavelengths and some broaden, and the estimated spectra were most consistent with measured spectra.

Supplementary Table 1: Plasmid construction scheme

Plasmid	Flourophore 1	Location 1	Flourophore 1	Location 2
**pCAG-EBFP**	EBFP2.0	Nucleolus	/	/
**pCAG-LSSmCherry -mClover3**	LSSmCherry	Whole cell	mClover3	Golgi apparatus
**pCAG -mKate- mCerulean**	mKate	Mitochondria	mCerulean	Cytoskeleton
**pCAG -TagBFP-mAmetine**	TagBFP	Nucleus	mAmetine	Endoplasmic reticulum

Supplementary Table 2: $\overline{SSIM}$ of different algorithms while changing the relative intensity of Fluo 1 and others from 0.1 to 10

	$\overline{SSIM}$					
**Relative intensity of Fluo 1 to others**	**LU**	**SIMI**	**NMF-RI**	**nNMF**	**PICASSO**	**BINGO**
0.1	0.7827	0.4498	0.7955	0.7508	0.8144	0.9267
0.2	0.8351	0.4813	0.8408	0.7504	0.8485	0.9366
0.3	0.8799	0.4819	0.8787	0.7819	0.8766	0.9452
0.4	0.8857	0.6131	0.8873	0.7911	0.8826	0.9496
0.5	0.9033	0.6454	0.8960	0.7921	0.9015	0.9506
0.6	0.9134	0.6387	0.9118	0.7880	0.9069	0.9600
0.7	0.9173	0.9077	0.9163	0.7841	0.9088	0.9630
0.8	0.9178	0.9160	0.9171	0.7821	0.9047	0.9625
0.9	0.9189	0.9172	0.9183	0.7796	0.8890	0.9490
1	0.8865	0.9173	0.8870	0.7788	0.8903	0.9489
2	0.8171	0.9083	0.8173	0.7920	0.8868	0.9462
3	0.7946	0.8971	0.7976	0.7957	0.8921	0.9389
4	0.7942	0.8709	0.7961	0.7952	0.8699	0.9081
5	0.7932	0.8570	0.7937	0.7760	0.8795	0.9364
6	0.7927	0.8499	0.7931	0.7754	0.8747	0.9357
7	0.7923	0.8471	0.7925	0.7745	0.8750	0.9348
8	0.7918	0.8414	0.7919	0.7741	0.8706	0.9348
9	0.7914	0.8319	0.7915	0.7738	0.8715	0.9424
10	0.7907	0.8285	0.7908	0.7734	0.8703	0.9421

Supplementary Table 3: $\overline{Dice}$ of different algorithms while changing the relative intensity of Fluo 1 and others from 0.1 to 10

	$\overline{Dice}$					
**Relative intensity of Fluo 1 to others**	**LU**	**SIMI**	**NMF-RI**	**nNMF**	**PICASSO**	**BINGO**
0.1	0.4589	0.1354	0.4639	0.4647	0.4680	0.8538
0.2	0.6929	0.1361	0.7042	0.4541	0.5953	0.8821
0.3	0.7415	0.1377	0.7353	0.6553	0.7745	0.8606
0.4	0.7784	0.4828	0.7785	0.7043	0.7939	0.8833
0.5	0.8291	0.5416	0.8177	0.7230	0.8218	0.8872
0.6	0.9178	0.5806	0.9052	0.7252	0.8697	0.9029
0.7	0.9412	0.8805	0.9422	0.7193	0.9163	0.9032
0.8	0.9419	0.9256	0.9432	0.6956	0.9510	0.9079
0.9	0.9296	0.9343	0.9299	0.5249	0.9363	0.9169
1	0.9186	0.9390	0.9190	0.5008	0.9334	0.9246
2	0.5402	0.9363	0.5402	0.4927	0.9700	0.9264
3	0.5301	0.8871	0.5429	0.4952	0.9700	0.9299
4	0.5285	0.8118	0.5410	0.4928	0.9607	0.9293
5	0.5238	0.7230	0.5301	0.4885	0.7302	0.9296
6	0.5212	0.6651	0.5272	0.4838	0.7244	0.9286
7	0.5222	0.6354	0.5241	0.4780	0.7232	0.9283
8	0.5202	0.6460	0.5205	0.4714	0.7118	0.9264
9	0.5173	0.6465	0.5174	0.4676	0.7121	0.9264
10	0.5139	0.6479	0.5140	0.4647	0.7084	0.9245

Supplementary Table 4: $\overline{SSIM}$ of different algorithms while changing the number of fluorophores from 3 to 11

Number of fluorophores	$\overline{SSIM}$					
	LU	SIMI	NMF-RI	nNMF	PICASSO	BINGO
3	0.4336	0.4970	0.6203	0.6138	0.6695	0.7478
4	0.4319	0.5939	0.5649	0.5073	0.6438	0.8226
5	0.4238	0.6184	0.5060	0.4609	0.6209	0.8526
6	0.4584	0.6460	0.5151	0.4493	0.6184	0.8958
7	0.4316	0.6562	0.4796	0.4142	0.5661	0.9039
8	0.4275	0.6561	0.4498	0.3917	0.5150	0.8913
9	0.4164	0.6757	0.4299	0.3739	0.5011	0.9025
10	0.4213	0.6836	0.4158	0.3611	0.4793	0.8954
11	0.4231	0.6913	0.4185	0.3651	0.4983	0.9070

Supplementary Table 5: $\overline{Dice}$ of different algorithms while changing the number of fluorophores from 3 to 11

Number of fluorophores	$\overline{Dice}$					
	LU	SIMI	NMF-RI	nNMF	PICASSO	BINGO
3	0.8793	0.8437	0.8824	0.7239	0.9058	0.8236
4	0.9678	0.8273	0.9725	0.7013	0.9487	0.9655
5	0.9627	0.8475	0.9611	0.6701	0.9659	0.9627
6	0.9705	0.8754	0.9674	0.6609	0.9674	0.9809
7	0.9677	0.8757	0.9500	0.6782	0.9538	0.9710
8	0.9850	0.8466	0.9147	0.6766	0.9754	0.9614
9	0.9809	0.8744	0.9210	0.6582	0.9777	0.9619
10	0.9629	0.8897	0.8971	0.6493	0.9432	0.9765
11	0.9665	0.8670	0.8782	0.6427	0.9497	0.9788

Supplementary Table 6: SSIM of different algorithms with 10-color fluorescent beads

Fluorescent beads	*SSIM*					
	**LU**	**SIMI**	**NMF-RI**	**nNMF**	**PICASSO**	**BINGO**
Blue	0.7275	0.7729	0.9304	0.9351	0.9193	0.9278
Blue-green	0.9574	0.9581	0.9587	0.9550	0.9595	0.9576
Green	0.9209	0.8850	0.9557	0.9609	0.9659	0.9659
Yellow-green	0.5253	0.4100	0.7271	0.5693	0.8423	0.8234
Yellow	0.9101	0.8971	0.9286	0.9410	0.9386	0.9466
Orange	0.9139	0.9294	0.9180	0.9382	0.9680	0.9698
Red	0.9269	0.4036	0.9202	0.9121	0.9371	0.9481
Red-orange	0.9321	0.9212	0.9608	0.9186	0.9138	0.9540
Crimson	0.5839	0.7990	0.6766	0.5998	0.7837	0.8699
Scarlet	0.1969	0.3210	0.6401	0.5670	0.6976	0.8699

Supplementary Table 7: Dice of different algorithms with 10-color fluorescent beads

Fluorescent beads	*Dice*					
	**LU**	**SIMI**	**NMF-RI**	**nNMF**	**PICASSO**	**BINGO**
Blue	0.7227	0.7237	0.5431	0.4815	0.6716	0.5935
Blue-green	0.6912	0.7284	0.8068	0.8058	0.8018	0.7952
Green	0.3187	0.0082	0.7337	0.8438	0.8379	0.8350
Yellow-green	0.0063	0.0094	0.0252	0.7419	0.3254	0.6047
Yellow	0.0000	0.0012	0.2589	0.9275	0.7163	0.8705
Orange	0.0020	0.0021	0.0315	0.7759	0.8761	0.8731
Red	0.0010	0.0180	0.0271	0.4643	0.3916	0.6167
Red-orange	0.6304	0.3297	0.7505	0.2155	0.0326	0.5863
Crimson	0.1441	0.0448	0.6436	0.7931	0.7988	0.9312
Scarlet	0.6355	0.6088	0.9292	0.7551	0.9457	0.9458

Reference

Rakhymzhan, A., *et al*. (2017) Synergistic Strategy for Multicolor Two-photon Microscopy: Application to the Analysis of Germinal Center Reactions In Vivo, *Sci Rep,* 7 (1), 7101.

Zimmermann, Timo (2005) Spectral Imaging and Linear Unmixing in Light Microscopy, In: Jens R. (ed.), *Microscopy Techniques.* Springer Berlin Heidelberg, Berlin, pp. 245-265.

Zimmermann, Timo, *et al*. (2014) Clearing Up the Signal: Spectral Imaging and Linear Unmixing in Fluorescence Microscopy, In: Stephen W. P. (ed.), *Confocal Microscopy: Methods and Protocols.* Springer New York, NY, pp. 129-148.
